# A smart ZnO@polydopamine-nucleic acid nanosystem for ultrasensitive live cell mRNA imaging by the target-triggered intracellular self-assembly of active DNAzyme nanostructures†
†Electronic supplementary information (ESI) available: Oligonucleotide sequences, DLS and zeta potential measurements, TEM images, absorption and fluorescence spectra, cytotoxicity assay and CLSM images. See DOI: 10.1039/c6sc04633a



**DOI:** 10.1039/c6sc04633a

**Published:** 2017-01-19

**Authors:** Dinggeng He, Xing He, Xue Yang, Hung-Wing Li

**Affiliations:** a Department of Chemistry , Hong Kong Baptist University , Kowloon Tong , Hong Kong , China . Email: hwli@hkbu.edu.hk; b State Key Laboratory of Chemo/Biosensing and Chemometrics , College of Chemistry and Chemical Engineering , Hunan University , Changsha 410082 , China

## Abstract

Efficient strategies for the ultrasensitive imaging of gene expression in living cells are essential in chemistry and cell biology. Here, we report a novel and efficient enzyme-free dual signal amplification strategy for live cell mRNA imaging by using a smart nucleic acid hairpin-based nanosystem. This nanosystem consists of a ZnO nanoparticle core, an interlayer of polydopamine and an outer layer of four hairpin DNA (hpDNA) probes. Such a core–shell nanosystem facilitates the cellular uptake of molecular hairpin payloads, protects them from nuclease digestion, and delivers them into the cytoplasm by the acid-triggered dissolution of the ZnO core. In the presence of target mRNA, the released hpDNA probes self-assemble *via* HCR into wire-shaped active DNAzymes that catalyze the generation of a fluorescence signal. The target-initiated HCR events and DNAzyme cascades offer efficient dual amplification and enable the ultrasensitive detection of mRNA with a femtomolar detection limit. Live cell assays show an intense fluorescence response from a tumor-related biomarker *survivin* mRNA only in tumor cells untreated with a *survivin* expression repressor YM155, but not in normal cells. The developed nanosystem provides a potential platform for the amplified imaging of low-abundance disease-related biomarkers in live cells.

## Abstract 2

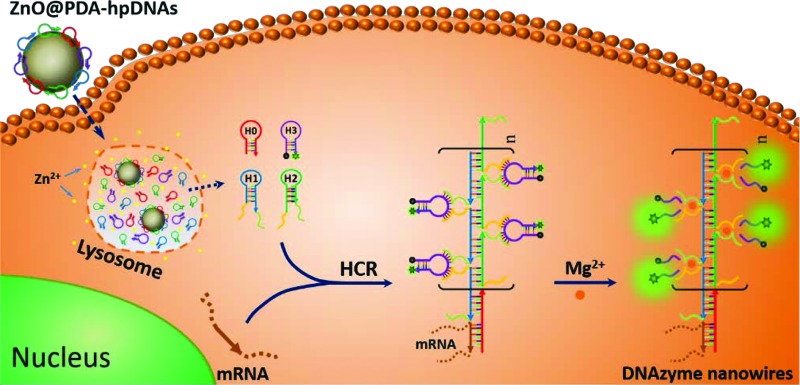
We have developed a smart ZnO@PDA-hpDNAs nanosystem carrying four functional hpDNA strands that can realize the target-triggered self-assembly of wire-shaped active DNAzyme nanostructures *via* HCR in live cells.

## Introduction

The fundamental understanding of cellular processes involving intracellular elementary biomolecules, especially disease-related biomarkers with low expression levels, is essential in cell biology, disease pathophysiology, drug discovery, medical diagnostics, and therapeutic applications.^1^ In view of this, ultrasensitive amplified techniques for the visualization and localization of cellular low-abundance biomarkers at the single-cell level have been developed. It is well-known that messenger ribonucleic acids (mRNAs) regulate and control the fate, function and phenotype of cells. Their abnormal expression probably causes disease. Thus, the sensitive detection and identification of mRNA have attracted substantial research efforts, and several amplified sensing techniques have been reported for intracellular mRNA expression, for instance, fluorescent *in situ* hybridization (FISH),^2^ polymerase chain reaction (PCR)^3^ and rolling circle amplification (RCA).^4^ However, these techniques require the extraction of mRNA from cells or the fixation of cells prior to analysis and thus are unable to perform real-time *in situ* monitoring of intracellular mRNA expression levels under *in vivo* conditions.

Recently, several *in vivo* hybridization techniques based on nucleic acid hairpin models have been developed, demonstrating novel enzyme-free amplified strategies for the optical detection and imaging of related mRNA inside live cells (the term “enzyme-free” refers to a protein-free catalyst). These nonenzymatic signal amplification mechanisms, such as the cascade hybridization reaction (CHR),^5^ cascade reaction^6^ and hybridization chain reaction (HCR),^7^ can significantly enhance the fluorescence signal produced by the primary hybridization events of hairpin DNA probes and targets. Despite the fact that these reports have featured the ultrasensitive imaging analysis of mRNA expression levels of live cells by enzyme-free amplification strategies, there is still a lack of effective nonenzymatic dual signal amplification techniques involving the use of catalytic nucleic acids (DNAzymes or ribozymes) for ultrasensitive live cell mRNA imaging.

**Scheme 1:**
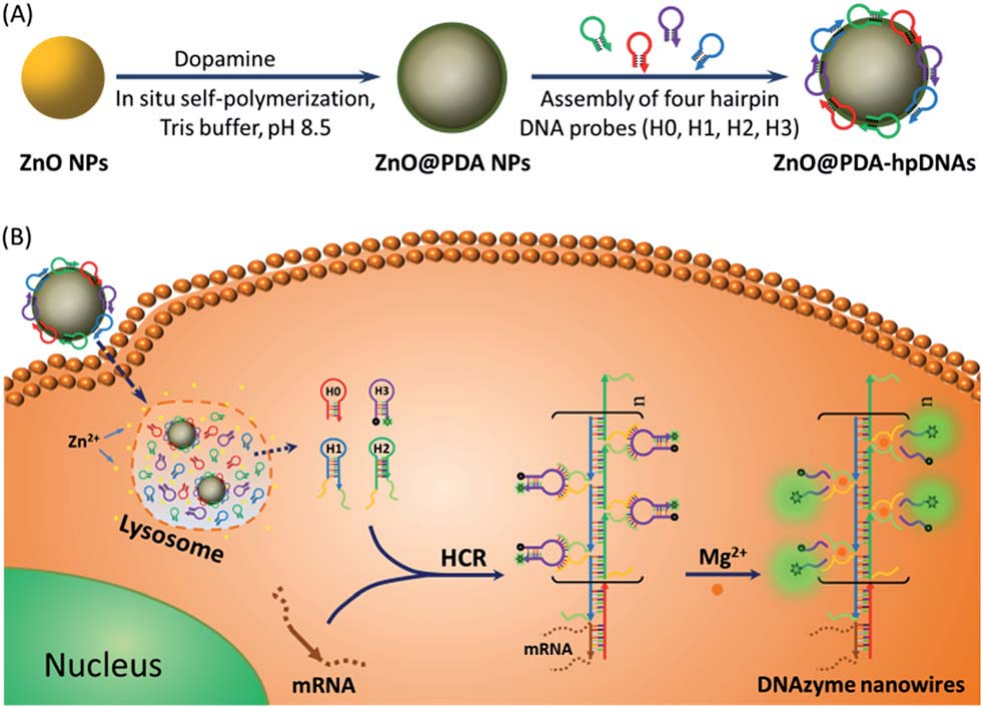
(A) Preparation of the polydopamine-coated ZnO nanoparticles (ZnO@PDA NPs) and functional hairpin DNA strands (hpDNAs)-immobilized ZnO@PDA (ZnO@PDA-hpDNAs) nanosystem. (B) Illustration of the ZnO@PDA-hpDNAs nanosystem for live cell mRNA imaging *via* the target-triggered HCR-mediated intracellular self-assembly of wire-shaped active DNAzyme nanostructures.

DNAzymes have been found to be of growing interest as amplifying techniques for analytical recognition events.^8^ The flexibility in regulating DNAzyme structure by encoding specific functional information in its base sequence turns DNAzyme into an ideal candidate for the development of live cell biosensing platforms. In this work, we have developed a novel DNAzyme-based enzyme-free dual amplification strategy for the fluorescence activation imaging of mRNA under *in vivo* conditions by using a smart hairpin-DNA-based nanosystem, which can facilitate the cellular uptake of molecular hairpins, protect them from nuclease digestion, and deliver them into the cytoplasm for the target-triggered self-assembly of wire-shaped DNA nanostructures consisting of numerous active DNAzyme subunits inside live cells. This smart nanosystem possesses a core of biodegradable ZnO nanoparticles (ZnO NPs), an interlayer of polydopamine (PDA) that is several nanometers thick deposited on the surface of ZnO *via in situ* self-polymerization under alkaline conditions, and an outer layer of four functional hpDNA probes (H0, H1, H2 and H3) immobilized onto the PDA simply by π–π interactions and hydrogen bonding,^9^ as illustrated in Scheme 1A. The resulting core–shell structured ZnO@PDA-hpDNAs nanosystem can easily enter cells by the cellular endocytic pathway without the aid of transfection agents.^10^ More importantly, after being engulfed by cells, the ZnO cores decomposed into Zn^2+^ ions in the acidic endosomes/lysosomes (pH 5.0–6.0) of the cells, leading to the dissociation of the ZnO@PDA-hpDNAs nanosystem and the delivery of immobilized hpDNA probes into the cytoplasm (Scheme 1B). In the presence of target mRNA, the specific binding between probe H0 and the target mRNA triggers the cross-opening of H1 and H2 in the cytoplasm of live cells, resulting in the formation of wire-shaped DNA nanostructures consisting of numerous tandem Mg^2+^-dependent DNAzyme subunits that can catalyze the cleavage of fluorescence self-quenching substrate H3 and the generation of a detectable fluorescent signal. The successful HCR events and DNAzyme cascades triggered by the target may enormously enhance the readout of the fluorescence signal and realize ultrasensitive amplified imaging for disease-related biomarkers with low copy numbers in living cells. To our best knowledge, this is the first time that the catalytic DNAzyme-based enzyme-free dual amplification strategy has been successfully achieved inside live cells by a rationally designed nucleic acid nanosystem. Using the developed nanosystem, we show the fluorescence imaging of *survivin* mRNA, an important mRNA known to be overexpressed in most cancer cells^11^ and in living cells of diverse types.

## Results and discussion

### Design of functional hpDNA probes and the target-triggered self-assembly of wire-shaped DNA nanostructures

**Fig. 1:**
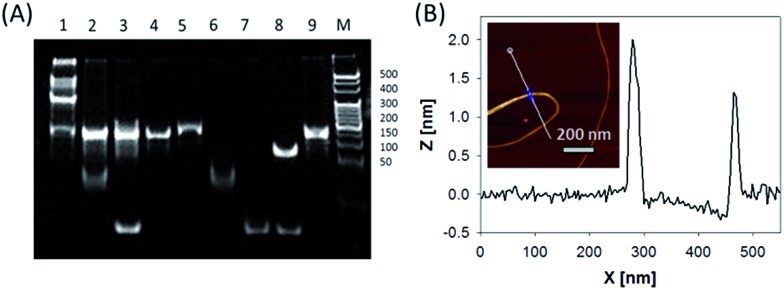
(A) Polyacrylamide gel electrophoresis: H0, H1 and H2 with the target sequence (lane 1); H0, H1 and H2 mixture (lane 2); H1 and H2 with the target sequence (lane 3); H1 (lane 4); H2 (lane 5); H0 (lane 6); target sequence (lane 7); H0 with the target sequence (lane 8); H1 and H2 mixture (lane 9). (B) AFM image and cross-section analysis of wire-shaped DNA nanostructures formed by the target-triggered HCR event.

In this system, the design of hairpin DNA (hpDNA) probes is the key to the success of the ultrasensitive imaging analysis experiments (Table S1 and Scheme S1†). The system consists of four functional hpDNA probes, namely H0, H1, H2 and H3. The hairpins H1 and H2 at their 5′ and 3′ ends, respectively, possess two separate single-strand tails with sequences of split Mg^2+^-dependent DNAzyme fragments with a “caged” and inactive structure. H0 is a recognition hairpin for the target sequence and, upon opening, it initiates the cross-opening of hairpins H1 and H2, resulting in the self-assembly of wire-shaped active DNAzyme nanostructures. Gel electrophoresis was performed to validate the successful formation of DNA nanostructures (Fig. 1A). H0, H1 and H2 showed good metastability individually (lanes 6, 4 and 5, respectively) and their mixture exhibited no reaction in the absence of the target (lanes 2 and 9). Lane 3, corresponding to a mixture of H1, H2 and the target, exhibited a lack of the HCR event. Lane 8 demonstrated only the hybridization reaction between H0 and the target in the absence of H1 and H2. Lane 1 showed ladder shaped bands when H0, H1, H2 and the target existed simultaneously, indicating successful HCR. Fig. 1B shows an atomic force microscopy (AFM) image of the resultant HCR production in which micrometer-long wire-shaped DNA nanostructures were observed. The height of the nanowires was in the range of 1.3–1.9 nm, which is almost the same as the width of double-stranded DNA. The formed DNA nanostructures with the assistance of Mg^2+^ ions could catalyze the cleavage of the ribonucleobase (rA)-containing substrate H3 that was modified at its 5′ and 3′ ends with a quencher/fluorophore pair, resulting in the activation of a fluorescence signal. Fig. S1A† displays the time-dependent fluorescence changes under different conditions. Fig. S1B† displays the fluorescence spectra of the system upon analysis of different concentrations of the target sequence. The resulting calibration curve is shown in Fig. S1B,† inset. The target sequence could be detected with a detection limit of 1 fM. To further investigate the effect of the HCR event on the detection sensitivity, a DNAzyme system without the HCR event was designed and performed for analysis of the target sequence (Scheme S2 and Fig. S2†). This HCR-deficient system showed a lower fluorescence signal than the HCR-based system under the same conditions, thus suggesting that HCR events enhance the detection sensitivity towards the target.

### Synthesis and characterization of the ZnO@PDA NPs and ZnO@PDA-hpDNAs nanosystem

The aforementioned smart nanosystem carrying four hpDNA probes was established by the introduction of biodegradable ZnO NPs as the carriers. This is because ZnO NPs are cost-effective nanomaterials of low toxicity and are able to decompose completely at pH 5.0 in aqueous solution.^12^ To further improve the immobilizing efficiency of the hpDNA probes, a biocompatible PDA shell was employed owing to its abundant catechol and amino groups, which allowed for the facile and direct immobilization of DNA strands *via* π–π interactions and hydrogen bonding. The introduction of PDA was performed by the *in situ* self-polymerization of the small molecule dopamine on the surface of ZnO NPs. To tune the coating amount of PDA, the commercially available ZnO NPs were ultrasonically dispersed in a Tris buffer solution (10 mM, pH 8.5) containing different concentrations of dopamine for 1 h. The coating amount of PDA was then determined by thermogravimetric measurements to be about ∼1%, ∼3% and ∼5% (mass percentage of PDA in ZnO@PDA). UV-vis absorption spectra of the ZnO@PDA NPs revealed an increase in near-infrared (NIR) absorbance with the increasing amount of PDA coating (Fig. S3†). The generation of the PDA shell resulted in a decrease in the surface charges of the nanoparticles, as demonstrated by zeta potential analysis (Table S2†). Dynamic light scattering (DLS) measurements showed the changes in the hydrodynamic diameters of the ZnO NPs before and after treatment with different concentrations of dopamine. It is worth noting that the hydrodynamic size of the nanoparticles and the thickness of the PDA shell can be easily tuned by simply changing the dopamine concentrations. The core–shell structure of the ZnO@PDA NPs was clearly visible in the transmission electron microscopy (TEM) images (Fig. S4†). The ZnO@PDA NPs (containing 3% PDA) possessed a desired PDA shell thickness of ∼8.6 ± 2.7 nm, which was in accordance with the result of the DLS measurement (∼8.4 ± 1.3 nm). Both the TEM and DLS data indicated that the ZnO@PDA NPs (containing 3% PDA) fell within a size range that favored cellular uptake by mammalian cells.^13^ Thus, the ZnO@PDA NPs containing 3% PDA were used in the following experiments.

To investigate the immobilizing behavior of the functional hpDNA probes, carboxyfluorescein (FAM)-labeled hpDNA H4 that can specifically recognize *survivin* mRNA (sequence listed in Table S1†) was incubated with the ZnO@PDA NPs for 1 h to form a ZnO@PDA-H4 nanosystem probe. The fluorescence spectra of H4 before and after being incubated with the ZnO@PDA NPs were then compared. Fig. S5A† shows that the fluorescence of H4, when incubated with the ZnO@PDA NPs, almost entirely disappeared. This is because the PDA shell quenches the fluorescence of the FAM-labeled hpDNA H4 deposited on its surface.^10,14^ The fluorescence quenching efficiency was estimated to be approximately 93%. These data confirmed the successful immobilization of H4. The quenched fluorescence of the ZnO@PDA-H4 nanosystem could be activated in the presence of the target sequence (Fig. S5B†), suggesting that the nanosystem could be directly used for target detection.^15^ To demonstrate the versatility of our ZnO@PDA NPs, we repeated the above immobilization process using tetramethylrhodamine (TMR)-labeled hpDNA H5 (sequence listed in Table S1†). Again, H5 was successfully immobilized onto the surfaces of the ZnO@PDA NPs (Table S3†). Taking both the H4 and H5 experimental data together, the immobilization was independent of the sequence of the hpDNA strands. The average immobilizing amount of hpDNA probes on the ZnO@PDA NPs was estimated to be ∼207 ± 21 molecules per particle (corresponding to a 1.4 μmol g^–1^ nanosystem) *via* decomposition of the ZnO core using acid.

Next, the dissolution of ZnO NPs triggered by a pH-change was investigated using UV-vis absorption spectroscopy. The UV-vis absorption at around 380 nm, which corresponded to the band gap of ZnO, became weaker as the solution pH changed from 7.4 to 5.0 (Fig. S6†). No visible absorption band at the wavelength of 380 nm was observed in the UV-vis spectra when the pH value of the solution was 5.0, indicating that the ZnO NPs decomposed completely at pH 5.0. To demonstrate the pH-triggered delivery of hpDNA probes immobilized on the ZnO@PDA NPs surface, the ZnO@PDA-H5 nanosystem was added into Tris buffer solutions of different pH values. After 0.5 h of incubation under acidic pH conditions, the quenched fluorescence of H5 by the PDA shell was recovered because of the dissolution of the ZnO core (Fig. S7A†). Meanwhile, the by-product PDA fragments in solution had an insignificant effect on the fluorescence of the released H5. The hpDNA strands released from the surfaces of the nanoparticles were still intact after treatment with acid, as confirmed by gel electrophoresis (Fig. S7B†).

### *In vitro* response of the ZnO@PDA-hpDNAs nanosystem

**Fig. 2:**
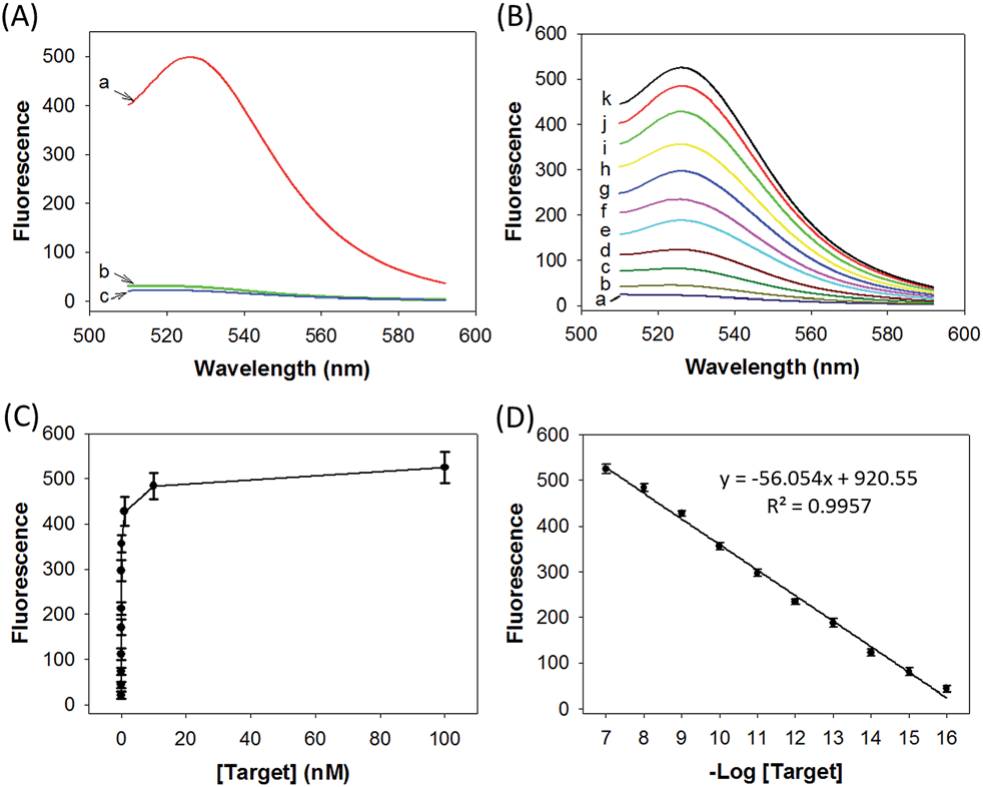
(A) Fluorescence spectra obtained by incubating the nanosystem carrying H0, H1, H2 and H3 with the (a) target sequence, (b) nontarget sequence, and (c) assay buffer as well as the target sequence. (B) Fluorescence spectral responses to the target sequence with different concentrations: (a) 0 M, (b) 1 × 10^–16^ M, (c) 1 × 10^–15^ M, (d) 1 × 10^–14^ M, (e) 1 × 10^–13^ M, (f) 1 × 10^–12^ M, (g) 1 × 10^–11^ M, (h) 1 × 10^–10^ M, (i) 1 × 10^–9^ M, (j) 1 × 10^–8^ M, and (k) 1 × 10^–7^ M target sequence. (C) Plot of fluorescence peak intensities (at 526 nm) *versus* target concentrations. (D) Fluorescence peak intensities *versus* target concentrations on a logarithmic scale.

We prepared a ZnO@PDA-hpDNAs nanosystem carrying four hpDNA probes, H0, H1, H2 and H3. This nanosystem pretreated with acid showed a particularly strong fluorescence response for the *in vitro* detection of the target sequence (Fig. 2A). An intense fluorescence peak was observed at a wavelength of 526 nm in the presence of a 10 nM target, giving a very high signal-to-background ratio of ∼25-fold in response to the target sequence. In contrast, no visible fluorescence peak appeared in the presence of a nontarget sequence. The nanosystem consisting of only three hpDNA probes, H1, H2 and H3, did not give an obvious fluorescence signal. In addition, we found that the nanosystem-based analysis had slower kinetics and a higher signal-to-background ratio when compared with that of the free hpDNA probes system (Fig. S8†). We anticipated that the slower kinetics was due to the interference of the by-product PDA fragments in solution, which might in a manner hinder the assembly of active DNAzyme nanostructures. The higher signal-to-background ratio that originated from a lower background signal will then enhance the detection sensitivity. The ZnO@PDA-hpDNAs nanosystem showed fluorescence signals that correlated to the concentrations of the target (Fig. 2B and C). A calibration curve was obtained for the fluorescence intensities at 526 nm for target concentrations (logarithmic scale) over a range from 0.1 fM to 100 nM, with a detection limit as low as 0.1 fM observed (Fig. 2D).

Afterwards, we investigated the stability of the ZnO@PDA-H4 nanosystem in PBS with high salt concentration as well as with a cell culture medium. The fluorescence data demonstrated that the ZnO@PDA-H4 nanosystem was stable in those solutions for 12 h upon incubation at 37 °C and showed no significant fluorescence changes (Fig. S9†). The nanosystem was also found to exhibit resistance to degradation by nuclease (Fig. S10†). Actually, no visible degradation of probe H4 on the surface of the nanosystem was observed in the presence of DNase I, whereas the self-quenching free probe H6 saw obvious nuclease-mediated degradation. This nuclease resistance was mainly attributed to the high steric hindrance concerning DNase I and its interactions with DNA, which verified the high-affinity assembly of the hpDNA probes on the PDA surface. These results implied the possibility of using this nanosystem in complicated biological systems.

To apply the developed nanosystem to live cell mRNA imaging, the cytotoxicity of the ZnO@PDA NPs was the major concern. An MTT assay was used for testing the toxicity of the ZnO@PDA NPs towards HeLa cells. ZnO@PDA NPs had no obvious cytotoxic effect on HeLa cells after 48 h of treatment, even at a concentration of 25 μg mL^–1^ (Fig. S11†). The cytotoxicity of the ZnO NPs at a concentration higher than 25 μg mL^–1^ was mainly ascribed to the intracellular dissolution of the ZnO NPs into Zn^2+^ ions.^16^ To prove this, we evaluated the cell viability of HeLa cells with comparable concentrations of Zn^2+^ ions (as ZnCl_2_) after incubation for 48 h. This sample showed strong cytotoxicity when the concentration exceeded 25 μg mL^–1^. The cytotoxicity of Zn^2+^ ions is due to the induction of reactive oxygen species (ROS) production, lipid peroxidation, and DNA damage.^17^ Furthermore, the PDA shell layer did not cause significant cytotoxicity,^18^ as proven by our cell viability data. Consequently, ZnO@PDA NPs at a low concentration have no effect on the cell viability of HeLa cells, indicating that ZnO@PDA NPs are appropriate nanocarriers for biomedical application.

### Cellular uptake and localization of the ZnO@PDA-hpDNA nanosystem

Further investigation into the cellular uptake of the ZnO@PDA-H7 nanosystem in HeLa cells was performed using Bio-TEM and confocal laser scanning microscopy (CLSM). A Bio-TEM image of the HeLa cell after incubation with the nanosystem at 37 °C for 1 h was obtained to prove the intracellular activity of the nanosystem. A representative TEM image (Fig. S12A†) clearly showed the presence of ZnO nanostructures (red arrows) in the cytosol, indicating that the nanosystem was effectively internalized into the cells. Upon further incubation with a nanosystem-free culture for another 10 h, no obvious ZnO nanostructures were observed in the cytosol of the previous cells (Fig. S12B†), suggesting that the internalized ZnO NPs were completely decomposed. CLSM images showed that green fluorescence of FAM was observed in the cytoplasm and localized especially in the lysosomes after 2 h of incubation (Fig. S13†). At the cellular level, the internalization of most nanoparticles occurs *via* the cellular endocytic pathway; the nanoparticles are trafficked into the early endosomes, then into the late endosomes/lysosomes, and finally fused with lysosomes. Both endosomes (pH 5.0–6.0) and lysosomes (pH 4.5–5.0) have an acidic microenvironment, which is distinct from that outside the cells (pH 7.4).^19^ Therefore, the majority of the internalized ZnO@PDA-H7 nanosystems dissociated because of the dissolution of the ZnO cores under the acidic conditions. Next, the released H7 probes diffused into the whole cells after incubation for another 2 h. In order to further confirm the intracellular dissolution of the ZnO cores into Zn^2+^ ions, zinquin ethyl ester, a zinc-specific fluorescent dye, was used to quantify the accumulation of Zn^2+^ ions in the cells. The cells with no agents showed weak fluorescence owing to the indispensable intracellular Zn^2+^ for cell growth (Fig. S14A†). In contrast, the cells incubated with ZnCl_2_ displayed bright colors (Fig. S14B†). In the cells treated with the ZnO@PDA-H7 nanosystem, the fluorescence was very strong, thus proving the biodegradation of the ZnO core (Fig. S14C†). It is noted that the fluorescence of zinquin ethyl ester is mainly concentrated in some regions that do not overlap completely with the regions showing fluorescence of FAM in the merged image. This is because the generated Zn^2+^ ions are enriched in the vesicles,^20^ while the released hpDNA is mainly located in the cytoplasm.

### Live cell mRNA imaging using the ZnO@PDA-hpDNAs nanosystem

**Fig. 3:**
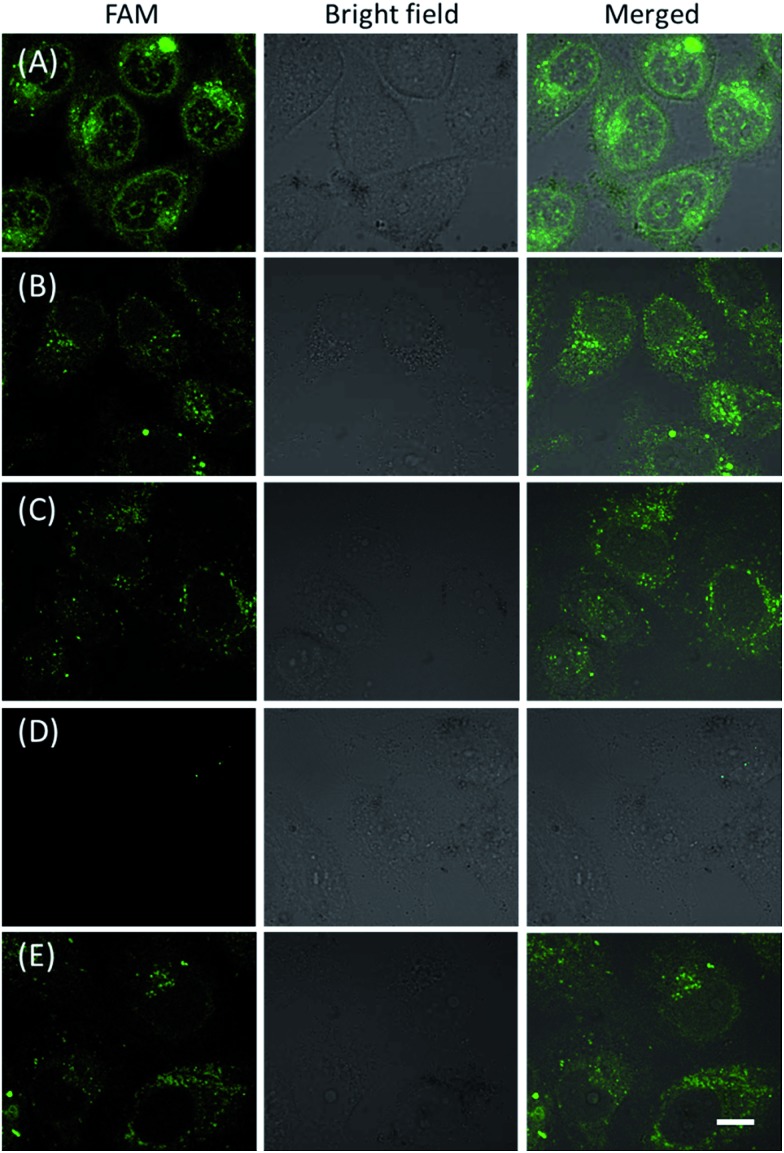
CLSM images of HeLa cells after being incubated with different nanosystems: (A) ZnO@PDA-hpDNAs nanosystem (10 μg mL^–1^), (B) ZnO@PDA-hpDNAs nanosystem (5 μg mL^–1^), (C) ZnO@PDA-H6 nanosystem (10 μg mL^–1^), (D) ZnO@PDA-H3 nanosystem (10 μg mL^–1^), and (E) ZnO@PDA-hpDNAs nanosystem (10 μg mL^–1^) without any additional Mg^2+^ ions. Scale bar = 25 μm.

The *in vitro* response and efficient cellular delivery of hpDNA probes provided the possibility for live cell mRNA imaging. After being incubated with the ZnO@PDA-hpDNAs nanosystem carrying the four hairpin probes, H0, H1, H2 and H3, HeLa cells showed a strong green fluorescence signal under excitation at 488 nm (Fig. 3A). The green fluorescence intensity is directly correlated with the concentration of the nanosystem (Fig. 3B). To obtain the desired intracellular fluorescence image, therefore, a final concentration of 10 μg mL^–1^ of the nanosystem was used in all of the following cell imaging. To confirm the sensitivity enhancement of the HCR- and DNAzyme-based amplification strategy, HeLa cells were incubated with the nanosystem that carries only the fluorescence self-quenching probe H6, which could directly hybridize with target mRNA and give a fluorescence signal in the cells. Fig. 3C shows low-contrast green fluorescence because of a lack of HCR events and active DNAzymes. In addition, HeLa cells that were incubated with the nanosystem carrying only the DNAzyme substrate H3 displayed insignificant fluorescence (Fig. 3D), suggesting that the substrate was stable in the absence of active DNAzyme. The fluorescence activation of substrate indicated the generation of active DNAzyme nanostructures, which is direct evidence of target mRNA expression and successful HCR in HeLa cells. Apart from the successful HCR, it was important that enough Mg^2+^ ions were present for the Mg^2+^-dependent DNAzyme-based fluorescence imaging of mRNA with sensitivity enhancement. Fig. 3E shows a much lower fluorescence signal when the cells were not pretreated with extra Mg^2+^ ions before incubation with the ZnO@PDA-hpDNAs nanosystem. The generation of a relatively weak fluorescence signal was attributed to the low intracellular catalytic activity of the DNAzymes, limited by the concentration of free Mg^2+^ ions in the cells (0.2–2 mM).^21^ Therefore, amplified fluorescence imaging of target mRNA was dependent on the abundance of the Mg^2+^ ions. This dual amplification strategy based on the target-triggered HCR and Mg^2+^-dependent DNAzyme could be a promising strategy for ultrasensitive mRNA imaging in living cells.

**Fig. 4:**
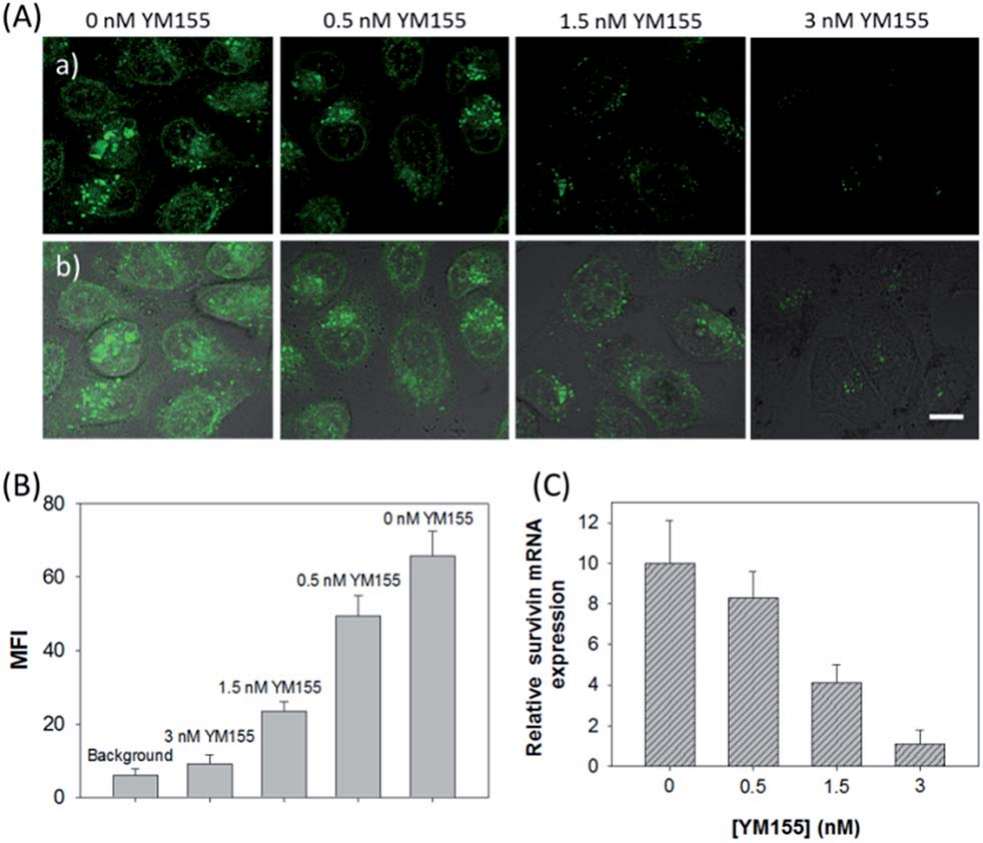
(A) CLSM images for HeLa cells treated with varying concentrations of YM155 followed by incubation with the ZnO@PDA-hpDNAs nanosystem (10 μg mL^–1^). (a) Green fluorescence images and (b) the merged images of fluorescence and bright field. Scale bar = 25 μm. (B) Fluorescence intensity comparison of the cells by flow cytometry. MFI represents the mean fluorescence intensity of intracellular FAM. (C) Relative expression levels of *survivin* mRNA in HeLa cells treated with varying concentrations of YM155.

Next, the developed nanosystem was applied for the quantitative analysis of the mRNA expression level in living cells. The intracellular *survivin* mRNA expression level was down-regulated by an imidazolium-based small-molecule compound YM155, a potent repressor of *survivin* mRNA expression.^22^ After being treated with YM155 of different concentrations, HeLa cells displayed a gradual decrease in fluorescence signal with the increasing dose of YM155 (Fig. 4A), indicating the successful inhibition of mRNA expression. The mean fluorescence intensities (MFI) of the cells after being treated with YM155 were measured using flow cytometry (Fig. 4B). The given fluorescence data showed a similar trend to that of the CLSM images. Subsequently, the relative expression level of *survivin* mRNA was quantified *via* normalization to an endogenous control of β-actin RNA. The fluorescence response upon adding the doses of YM155 was dynamically correlated to the relative levels of *survivin* mRNA, which were determined by the qualitative reverse transcription polymerase chain reaction (qRT-PCR) (Fig. 4C). In order to further investigate the sensitivity of mRNA detection in living cells, the total cellular RNA was extracted from the HeLa cells after being treated with different concentrations of YM155.^1c,7b^ Then, quantification of *survivin* mRNA levels in the cells was carried out by qRT-PCR (Table S4†). A calibration curve was constructed by plotting the MFI of the cells as a function of the target mRNA concentration. The linear range was determined as 0.01–3.85 amol ng^–1^ RNA, with a coefficient of 0.975. The limit of detection (LOD) was estimated to be 0.003 amol ng^–1^ RNA (or 16 copies per cell), suggesting that the developed strategy is an ultrasensitive approach for the imaging and analysis of intracellular mRNA in living cells.

**Fig. 5:**
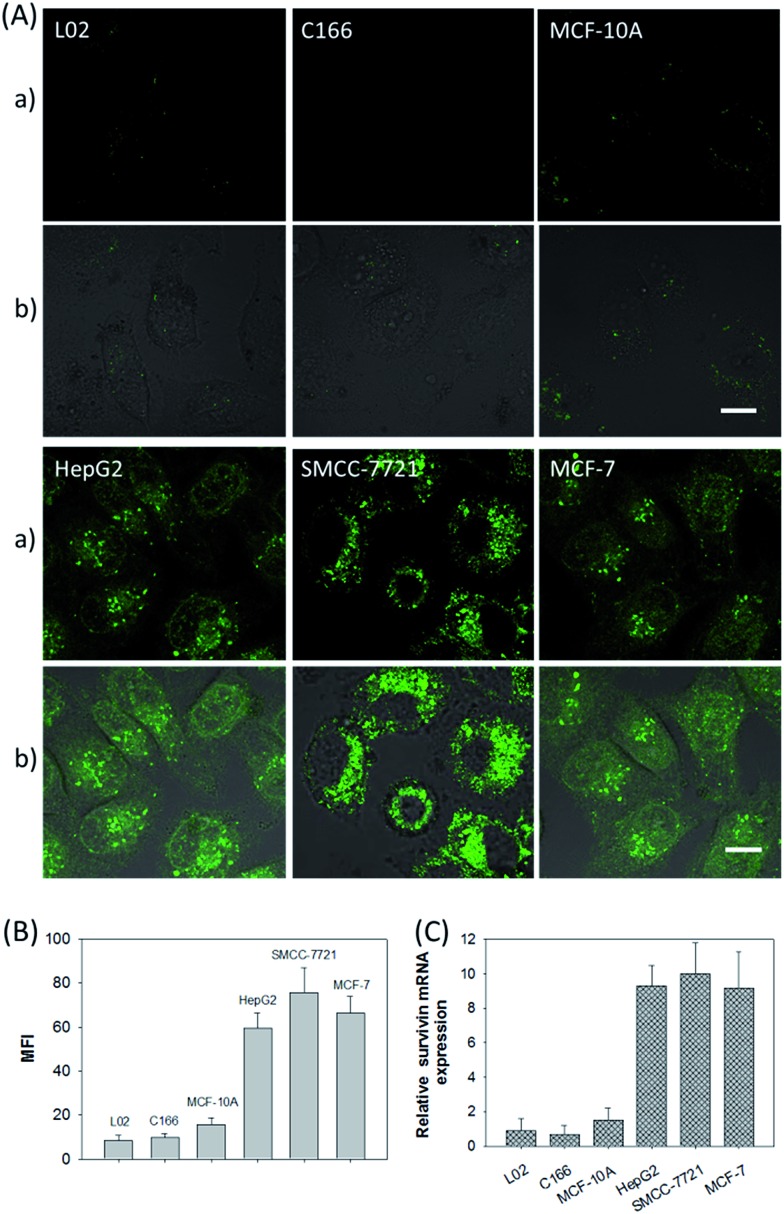
(A) CLSM images of different cells incubated with the ZnO@PDA-hpDNAs nanosystem (10 μg mL^–1^). (a) Green fluorescence images and (b) the merged images of fluorescence and bright field. Scale bar = 25 μm. (B) Fluorescence intensity comparison of the different cells by flow cytometry. (C) Relative expression levels of *survivin* mRNA in the L02, C166, MCF-10A, HepG2, SMCC-7721, and MCF-7 cells.

In addition, the relative *survivin* mRNA expressions in various cell lines, L02, C166, MCF-10A, HepG2, SMCC-7721, and MCF-7 cells, were also investigated using the developed nanosystem, as displayed in Fig. 5A. After being incubated with the ZnO@PDA-hpDNAs nanosystem, the L02 and MCF-10A cells showed merely a very weak fluorescence and the C166 cells showed no fluorescence activation, suggesting a low or absent expression of *survivin* mRNA. In contrast, the HepG2, SMCC-7721 and MCF-7 cells showed strong fluorescence signals, implying the high expression of target mRNA and the successful formation of active DNAzyme nanostructures in these cells. The MFI of the various cells was also determined by flow cytometry (Fig. 5B). Moreover, the relative concentrations of *survivin* mRNA in these cells were analyzed by the qRT-PCR method (Fig. 5C). These results were consistent with previous reports on the relative expression levels of *survivin* mRNA in various cells.^23^ In short, the developed nanosystem had a great potential application in quantitative mRNA imaging in living cells of diverse types.

## Experimental

### Chemicals and materials

Tris(hydroxymethyl)aminomethane hydrochloride (Tris), dopamine hydrochloride, 3-[4,5-dimethylthialzol-2-yl]-2,5-diphenyltetrazolium bromide (MTT), and *survivin* expression repressor YM155 were purchased from Sigma-Aldrich. ZnO colloidal solution (ZnO NPs, 50%) was purchased from Alfa Aesar. Zinquin ethyl ester was obtained from Enzo Life Sciences. LysoTracker Blue was obtained from Invitrogen. Nanopure water was used in all experiments and to prepare all buffers. All the chemicals were used as received without further purification. All oligonucleotides in this study were purchased from Shanghai Biotechnology Co. Ltd. and used without further purification. The human cervical carcinoma cell line (HeLa) and mouse endothelial cell line (C166) were obtained from the Cell Center of the Xiangya Medical School. The human breast adenocarcinoma cell line (MCF-7), immortalized nontumorigenic human mammary epithelial cell line (MCF-10A), hepatocellular carcinoma cell lines (SMCC-7721 and HepG2) and normal human hepatocyte cell line (L02) were purchased from the Shanghai Cell Bank, Chinese Academy of Medical Sciences.

### Hybridization assay of the probe and target

The *survivin* mRNA was chosen as the model target mRNA. Its DNA analogue was used for the following *in vitro* response experiments. Sequences of the oligonucleotides are listed in ESI Table S1.† Each functional hairpin DNA (1 μM) was heated to 95 °C for 5 min and then allowed to cool down to room temperature (25 °C) for at least 2 h before use. Then, the target sequence with variable concentration was added to 10 mM HEPES buffer containing 500 mM NaCl, 20 mM MgCl_2_ and a mixture of hairpin DNA (100 nM H0, 500 nM H1, 500 nM H2 and 500 nM H3). The time-dependent fluorescence changes upon adding different concentrations of target sequence were monitored at 37 °C using a fluorescence spectrofluorometer with excitation at 488 nm. For the AFM characterization of the wire-shaped DNA nanostructures that were produced by cross-opening of the functional DNA hairpin strands (H0, H1 and H2), the concentration of both hairpins was 500 nM and that of the target sequence was 10 nM. For polyacrylamide gel electrophoresis, the concentration of the target sequence was the same as that of the other hairpins (200 nM) to improve the quality of the image.

### Synthesis and characterization of the ZnO@PDA NPs

The commercially available ZnO NP stock solution was washed and dispersed in the Tris buffer (10 mM, pH 8.5). To coat the ZnO core with a PDA shell that was several nanometers thick, 1 mL of ZnO NPs (1 mg mL^–1^) solution was added into 10 mL of the freshly prepared Tris buffer solutions containing different concentrations of dopamine (5, 10 and 20 μg mL^–1^) under continuous sonication for 1 h at room temperature. The crude PDA-coated ZnO NPs (ZnO@PDA NPs) were purified by repeated centrifugation at 15 000 rpm for 10 min. The obtained ZnO@PDA NPs were redispersed in Nanopure water. The hydrodynamic size and zeta potential of the ZnO NPs before and after the PDA coating process were measured by a Nano ZS90 laser particle analyzer. UV-vis absorption spectra of the ZnO NPs and ZnO@PDA NPs solutions were recorded with a UV-2600 UV-vis spectrophotometer. The nanoparticles were also imaged by a JEOL 3010 microscope with an accelerating voltage of 100 kV. The amounts of PDA on the ZnO surfaces were determined by thermal gravimetric analysis using a TG 209 F1 (NETZSCH) instrument.

### Synthesis of the ZnO@PDA-hpDNAs nanosystem

In a typical synthesis, the smart nanosystem was prepared *via* the immobilization of functional hairpin DNA (hpDNA) on the surface of the ZnO@PDA NPs. To achieve this, 1 mL of ZnO@PDA NPs (0.1 mg mL^–1^) was mixed with 50 μL of each functional hpDNA (10 μM) under vortex agitation at room temperature for 1 h. The mixture was centrifuged at 15 000 rpm for 10 min to remove excess hpDNA strands. The sediments (ZnO@PDA-hpDNAs) were suspended in 1 mL of HEPES buffer (10 mM, pH 7.4) containing 500 mM NaCl and 20 mM MgCl_2_. Subsequently, the tetramethylrhodamine (TMR)-labeled hpDNA strands (H5) were used to determine the immobilizing amount of hpDNA strands on the surfaces of the ZnO@PDA NPs. In brief, the H5-immobilized ZnO@PDA NPs (ZnO@PDA-H5) were treated for 0.5 h with Tris buffer solutions with different pH values. After centrifugation at 15 000 rpm for 10 min, the fluorescence spectra of the supernatant were recorded using an F-7000 spectrofluorometer with excitation at 541 nm. The loading amount of the hpDNA strands was calculated to be 1.4 μmol g^–1^ of nanosystem.

### *In vitro* detection of the target

The DNA analogue of the *survivin* mRNA recognition sequence was used as the target for the extracellular studies. The ZnO@PDA-hpDNAs nanosystem (2 mg) consisting of four functional hpDNAs (H0, H1, H2 and H3) was added into 0.1 mL of Tris buffer solution (pH 5.0) and pretreated for 0.5 h. Subsequently, the resulting solution was mixed with 0.5 mL of HEPES buffer (10 mM, pH 7.4) containing 500 mM NaCl, 20 mM MgCl_2_ and different concentrations of the target, and then allowed to hybridize at 37 °C for 2 h. The mixture solution was immediately subjected to fluorescence measurements. Fluorescence spectra were recorded using the F-7000 spectrofluorometer with excitation at 488 nm.

### Stability testing of the nanosystem

The nuclease digestion assay for the nanosystem was performed. 10 μL of the nanosystem (100 μg mL^–1^) only carrying H4 was first mixed with 90 μL of Tris buffer (10 mM, pH 7.4) containing 25 mM MgCl_2_ and 5 mM CaCl_2_. 10 μL of DNase I (10 U L^–1^) was then added. The mixture was incubated at 37 °C for 2 h, and the fluorescence intensity was measured with 488 nm excitation and 526 nm emission. As a control, the fluorescence-quenched H6 with a final concentration of 14 nM (corresponding concentration to the H4 content of 10 μg mL^–1^ ZnO@PDA-H4) was incubated with DNase I under the same conditions.

### Cellular viability assay

HeLa cells were incubated in 96-well microplates with 200 μL of RPMI 1640 culture medium at a density of 1 × 10^4^ cells per well. After 24 h of incubation at 37 °C in a 100% humidified atmosphere containing 5% CO_2_, half of the culture medium was removed, and RPMI 1640 (100 μL) containing ZnO@PDA NPs or ZnCl_2_ with various concentrations were added in each well. The cells were incubated for 48 h. Subsequently, MTT solution (0.5 mg mL^–1^) was added to each well and the cells were incubated for another 4 h. The MTT medium was removed and 200 μL of DMSO was added to each well. The optical density (OD) was measured at 570 nm with a microplate reader. The cell viability was calculated as follows: viability = (OD_treated_/OD_control_) × 100%, where OD_treated_ was obtained from the cells treated with nanoparticles or other reagents and OD_control_ was obtained from the cells without any treatments.

### Cellular uptake and co-localization studies

HeLa cells were incubated with RPMI 1640 medium containing 10% fetal bovine serum (FBS) at 37 °C in a humidified atmosphere containing 5% CO_2_. The cells were seeded in a glass bottom dish and incubated in RPMI 1640 (2 mL) containing 10% FBS for 24 h. Thereafter, the ZnO@PDA-H7 nanosystem (10 μg mL^–1^) dispersed in RPMI 1640 medium with 10% FBS was added and the cells were further incubated at 37 °C for 2 and 4 h. For the co-localization study, 1 mL of LysoTracker Blue (25 nM) was added for the specific staining of the lysosomes of living cells for 15 min. After removing the medium and then washing with PBS (2 mL, pH 7.4), the cells were observed using a Fluoview FV500 (Olympus). In addition, to confirm the successful dissolution of the ZnO core and the accumulation of Zn^2+^ ions, zinquin ethyl ester (25 μM), a zinc-specific fluorescent dye, was used to treat the cells for 0.5 h prior to imaging.

Transmission electron microscopy (TEM) analysis was carried out to investigate the cellular uptake of the nanosystem. HeLa cells were first incubated with the ZnO@PDA-H7 nanosystem for 1 h. The cells were then incubated with a nanosystem-free culture for another 10 h. The trypsinized cells before and after being incubated with the nanosystem-free culture were fixed in 2.5% glutaraldehyde for 2 h, followed by washing with PBS, and post-fixing with 2% osmium tetroxide for 1 h. After that, the cells were dehydrated with acetone, embedded in Araldite resin, and sectioned with an ultramicrotome. The prepared ultrathin sections (70 nm) were collected on carbon-coated grids and imaged by a JEOL 3010 microscope with an accelerating voltage of 80 kV.

### Live cell mRNA imaging

HeLa cells were seeded in 35 mm culture dishes and incubated in 1 mL of RPMI 1640 medium containing 10% fetal bovine serum (FBS) at 37 °C in a humidified atmosphere containing 5% CO_2_ for 24 h. The various nanosystems (10 μg mL^–1^) were added to the medium and incubated at 37 °C for another 5 h. After washing three times with PBS, the cells were incubated with 1 mL of fresh medium at 37 °C before imaging. To downregulate the *survivin* mRNA expression in living cells, HeLa cells were first pretreated at 37 °C for 48 h with YM155 of a given concentration (0.5, 1.5 and 3.0 nM). Then, the cells were washed and incubated with the ZnO@PDA-hpDNAs nanosystem (10 μg mL^–1^) carrying four functional hpDNA probes (H0, H1, H2 and H3) at 37 °C for 5 h. CLSM images were obtained using a Fluoview FV500 (Olympus). Flow cytometry analyses were performed using a FACSCalibur flow cytometer (Becton Dickinson, San Jose, CA, USA), equipped with a 15 mW argon ion laser emitting at 488 nm for excitation. Quantitative reverse transcription-PCR (qRT-PCR) assays of *survivin* mRNA in cells were conducted according to the previously reported literature.^1c,7b^ In brief, the total cellular RNA of the HeLa cells was carefully extracted using an RNeasy Mini Kit (Qiagen, USA). The cDNA samples were then prepared by using a reverse transcription (RT) reaction with an iScript kit (Bio-Rad, USA). qPCR analysis of cDNA was performed with a SybrGreen PCR Master Mix (ABI, USA) according to the manufacturer's instructions. The *survivin* mRNA quantifications of the cells after treatment with different concentrations (0.5, 1.0, 1.5, 2.0, 2.5 and 3.0 nM) of YM155 were then determined.

The relative expression levels of *survivin* mRNA in various cell lines, such as the HepG2, L02, MCF-7, MCF-10A, SMCC-7721, and C166 cells, were investigated using the developed nanosystem. The HepG2, L02 and MCF-7 cells were incubated in RPMI 1640 culture medium with 10% FBS. The MCF-10A, SMCC and C166 cells were cultured in Dulbecco's-modified Eagles medium (DMEM) containing 10% FBS. All cell lines were maintained at 37 °C in a 100% humidified atmosphere containing 5% CO_2_. Then, these cells were incubated with the ZnO@PDA-hpDNAs nanosystem (10 μg mL^–1^) at 37 °C for 5 h. CLSM images were obtained using a Fluoview FV500. Flow cytometry analyses were performed using a FACSCalibur flow cytometer.

## Conclusions

In summary, we have developed a smart ZnO@PDA-hpDNAs nanosystem carrying four functional hpDNA strands that can realize the target-triggered self-assembly of wire-shaped active DNAzyme nanostructures *via* HCR in live cells. These intracellular target-initiated HCR events and DNAzyme cascades provide efficient dual signal amplification and enable the ultrasensitive detection of mRNA with a detection limit at the femtomolar level. The ZnO@PDA-hpDNAs nanosystem can be facilely prepared by the *in situ* self-polymerization of dopamine small molecules and subsequent immobilization of the mixture of hpDNA probes. The obtained core–shell structured nanosystem has a high immobilization efficiency for molecular hairpin probes, good biocompatibility and high resistance to DNase-mediated degradation. Moreover, the nanosystem facilitates the cell internalization of the DNA probes *via* an endocytic pathway by means of the acid-triggered degradation of nanocarriers and the stimuli-responsive delivery of probes. Live cell assays indicate that the developed nanosystem is very selective and ultrasensitive to the target mRNA. Consequently, the smart nanosystem provides an invaluable sensing platform for the ultrasensitive fluorescence imaging analysis of disease-related biomarkers with low expression levels inside live cells.
