# Supplementary material for: A smart ZnO@polydopamine-nucleic acid nanosystem for ultrasensitive live cell mRNA imaging by the target-triggered intracellular self-assembly of active DNAzyme nanostructures
Source: Chem Sci. 2017 Jan 19;8(4):2832–40. doi: 10.1039/c6sc04633a (PMC5427684; doi:10.1039/c6sc04633a)
Supplement: Supplementary file 1 [file SC-008-C6SC04633A-s001.pdf]

Electronic Supplementary Material (ESI) for Chemical Science.
This journal is © The Royal Society of Chemistry 2017

A smart ZnO@polydopamine-nucleic acid nanosystem for ultrasensitive live cell mRNA imaging by the target-triggered intracellular self-assembly of active DNAzyme nanostructures

Dinggeng He,[a,b] Xing He,[b] Xue Yang[b] and Hung-Wing Li*,[a]

[a] Department of Chemistry, Hong Kong Baptist University, Kowloon Tong, China

[b] State Key Laboratory of Chemo/Biosensing and Chemometrics, College of Chemistry and Chemical Engineering, Hunan University, Changsha 410082, China

*To whom correspondence should be addressed. Email: hwli@hkbu.edu.hk

Table S1 Sequences of oligonucleotides used in this work

Probe	RNA or DNA sequence (5'-3')
Target RNA	UCU CAA GGA CCA CCG CAU CUC UAC
DNA analogue of target RNA	TCT CAA GGA CCA CCG CAT CTC TAC
Nontarget DNA	TAG CTT ATC AGA CTG ATG TTG AAT
Hairpin probe H0	ACG TAG AGA TGC GGT GGT CCT TGA GAC ATA AGA CTT CTA GCA TCT CTA CGT
Hairpin probe H1	CCT CTC GAT ATC AGC GAT CTT CTA GCA TCT CTA CAA GTT ATT AAG TAG AGA TGC TAG AAG TCT TAT GAA GCA CCC ATG TTA CTC TCC C
Hairpin probe H2	CCT CTC GAT ATC AGC GAT CTT TTA ATA ACT TGT AGA GAT GCT AGC ATA AGA CTT CTA GCA TCT CTA CAA GCA CCC ATG TTA CTC TCC C
Hairpin probe H3	BHQ1- GCA GCA GAA AAA AAA AAA GGG AGA GTA TrAG GAT ATC GAG AGG AAA AAA AAA ATC TGC TGC -FAM
Hairpin probe H4	FAM- ACG TAG AGA TGC GGT GGT CCT TGA GAC ATA AGA CTT CTA GCA TCT CTA CGT
Hairpin probe H5	TMR- CCT CTC GAT ATC AGC GAT CTT CTA GCA TCT CTA CAA GTT ATT AAG TAG AGA TGC TAG AAG TCT TAT GAA GCA CCC ATG TTA CTC TCC C
Hairpin probe H6	BHQ1- ACG TAG AGA TGC GGT GGT CCT TGA GAC ATA AGA CTT CTA GCA TCT CTA CGT -FAM
Hairpin probe H7	FAM- TCA ATT TGA AGT CAA CAT CAG TCT GAT AAG CTA CAT AAG ACT TCT AAT TGA
DNAzyme 1 (D1)	CCT CTC GAT ATC AGC GAT CTT TGG TCC TTG AGA
DNAzyme 2 (D2)	GTA GAG ATG CGG AAG CAC CCA TGT TAC TCT CCC

H0 is a recognition hairpin probe that, after hybridizing with target RNA or its DNA analogue, has a single-strand tail to initiate the hybridization chain reaction between probes H1 and H2. H3 is the ribonucleobase (rA)-containing substrate that is modified at its 5' and 3' ends with a quencher/fluorophore (Q/F) pair, resulting in quenching of the luminescence of the fluorophore. H4 is carboxyfluorescein (FAM) labeled version of probe H0 at its 5' end. H5 is tetramethylrhodamine (TMR) labeled version of probe H1 at its 5' end. H6 is a self-quenched hairpin probe H0, which is used for testing the nuclease resistance of the nanosystem and validating the signal amplification in intracellular hybridization chain reaction. H7 is FAM labeled control probe at its 5' end, which is also

used for validating the intracellular acid-triggered delivery of hairpin DNA strands.

Table S2 Physiochemical properties of ZnO NPs and ZnO@PDA NPs

Sample	Hydrodynamic size [nm] (PDI)	Zeta potential [mV]
ZnO NPs	45.4 ± 3.2 (0.15)	-17.4 ± 2.5
ZnO@PDA NPs (1% PDA)	48.6 ± 5.1 (0.19)	-21.6 ± 1.8
ZnO@PDA NPs (3% PDA)	53.8 ± 4.5 (0.23)	-25.3 ± 2.7
ZnO@PDA NPs (5% PDA)	62.1 ± 6.3 (0.36)	-30.5 ± 3.6

Table S3 Physiochemical properties of ZnO@PDA-H5 NPs

Sample	Hydrodynamic size [nm] (PDI)	Zeta potential [mV]	Number of DNA per particle
ZnO@PDA NPs (3% PDA)	53.8 ± 4.5 (0.23)	-25.3 ± 2.7	—
ZnO@PDA-H5 (3% PDA)	61.3 ± 6.7 (0.18)	-18.5 ± 3.1	207 ± 21

Table S4 The *survivin* mRNA quantification of HeLa cells after being treated with YM155.

Sample	1	2	3	4	5	6
YM155 (nM)	0	0.5	1	1.5	2.5	3
Target mRNA (amol ng^{-1} RNA)	3.85	0.87	0.29	0.09	0.03	0.01

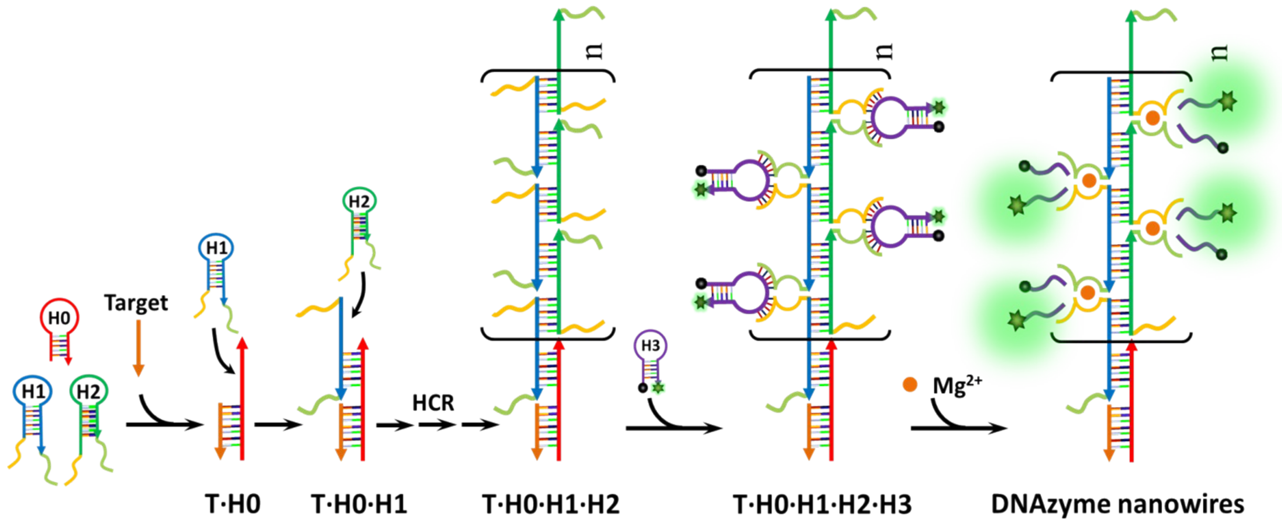

Scheme S1 Illustration of amplified analysis of the target sequence using four functional hairpin DNA probes (H0, H1, H2 and H3) for the target-initiated self-assembly of wire-shaped DNA nanostructures consisting of numerous tandem active Mg^{2+}-dependent DNAzyme subunits.

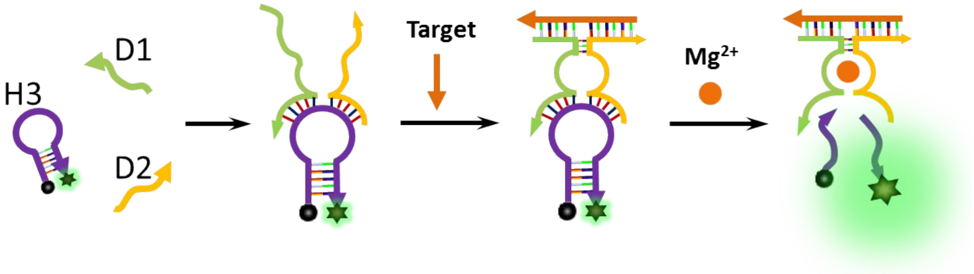

Scheme S2 Illustration of analysis of target sequence by using two split Mg^{2+}-dependent DNAzyme fragments (D1 and D2).

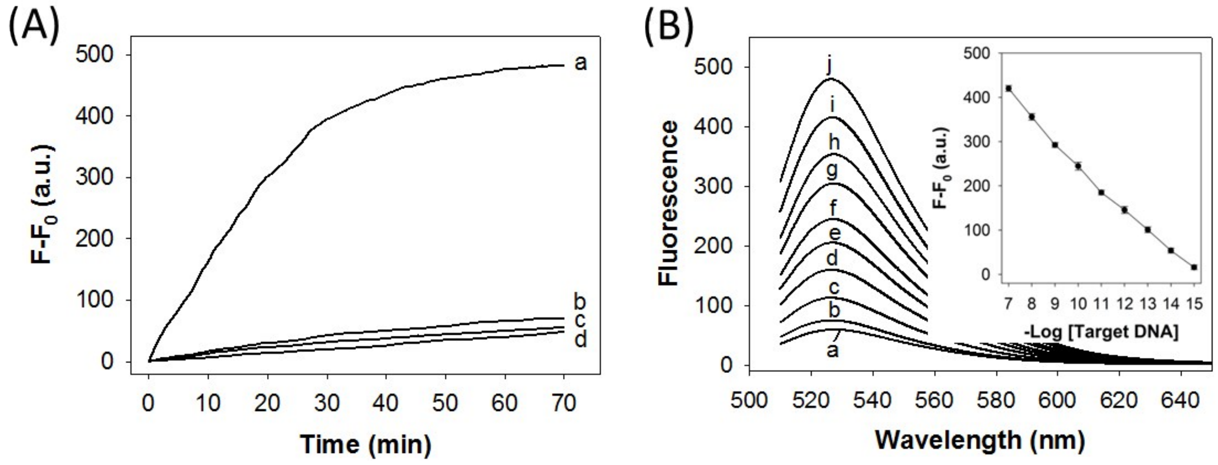

Fig. S1 (A) Time-dependent fluorescence changes under different conditions: a) H0, H1, H2, H3, target sequence and Mg^{2+}; b) H0, H1, H2, H3, nontarget sequence and Mg^{2+}; c) H1, H2, H3, target sequence and Mg^{2+}; d) H0, H1, H2, H3 and Mg^{2+}. (B) Fluorescence spectra corresponding to the analysis of different concentrations of target sequence: (a) 0 M, (b) 1×10^{-15} M, (c) 1×10^{-14} M, (d) 1×10^{-13} M, (e) 1×10^{-12} M, (f) 1×10^{-11} M, (g) 1×10^{-10} M, (h) 1×10^{-9} M, (i) 1×10^{-8} M, and (j) 1×10^{-7} M target sequence. Inset: Calibration curve corresponding to the fluorescence intensities at the wavelength of 526 nm in the presence of target sequence with different concentrations (logarithmic scale).

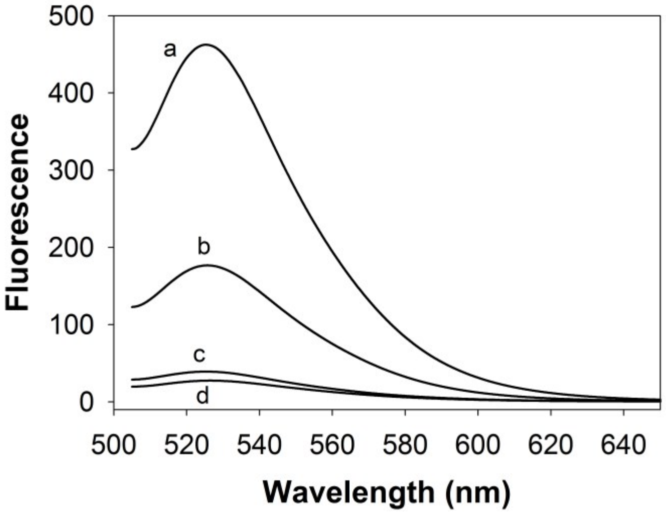

Fig. S2 Fluorescence spectra under different conditions: (a) H0, H1, H2, H3, target sequence and Mg^{2+}; (b) D1, D2, H3, Mg^{2+} and target sequence; (c) H0, H1, H2, H3 and Mg^{2+}; (d) D1, D2, H3 and

Mg^{2+}.

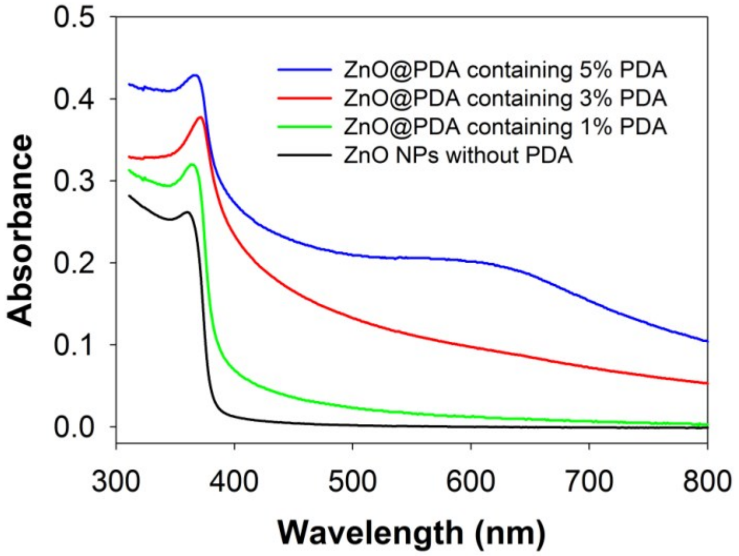

Fig. S3 UV-vis absorption spectra of aqueous solutions of ZnO NPs and ZnO@PDA NPs containing different amounts of PDA.

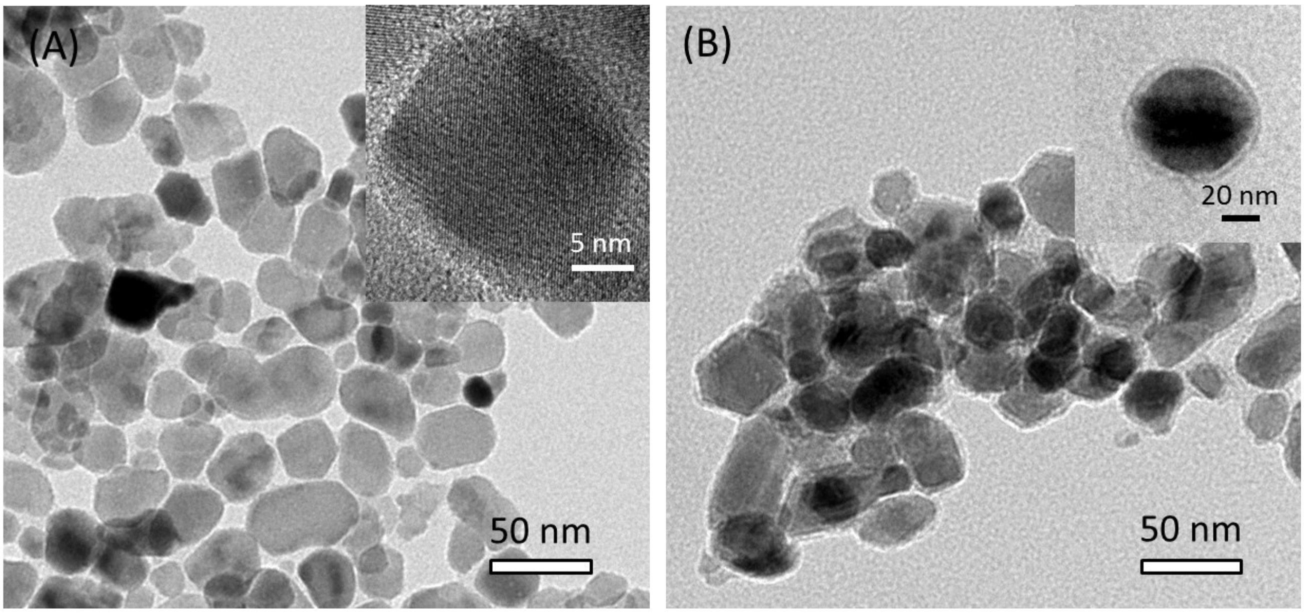

Fig. S4 TEM images of ZnO NPs (A) and ZnO@PDA NPs (B). Inset: HRTEM images of ZnO NPs (A) and ZnO@PDA NPs (B).

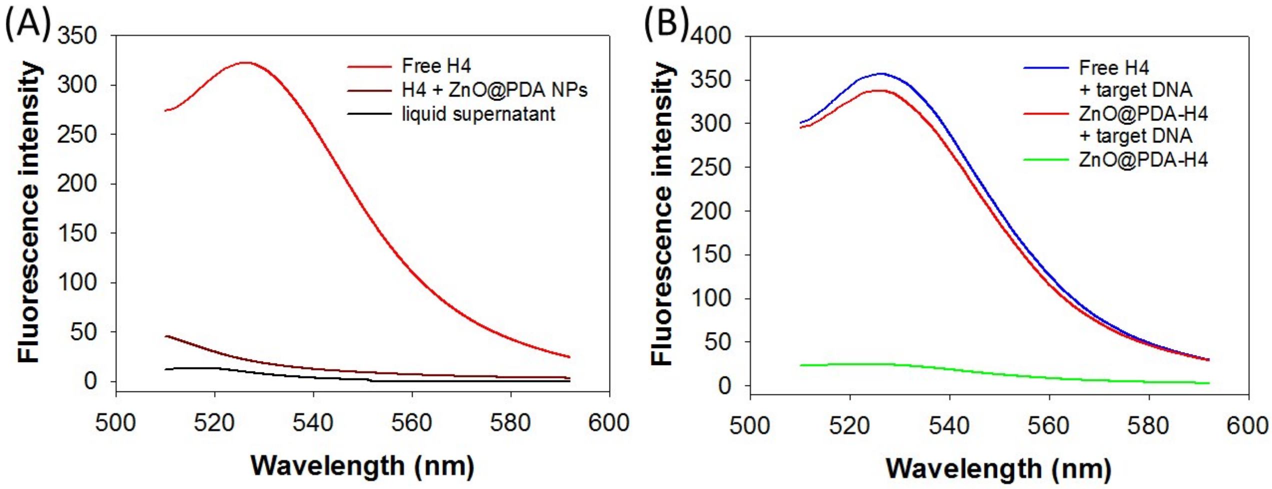

Fig. S5 (A) Fluorescence spectra of H4 (20 nM) before and after the addition of ZnO@PDA NPs. (B) Fluorescence spectra of ZnO@PDA-H4 nanosystem and free H4 (20 μg mL^{-1}) in the presence of target sequence (50 nM).

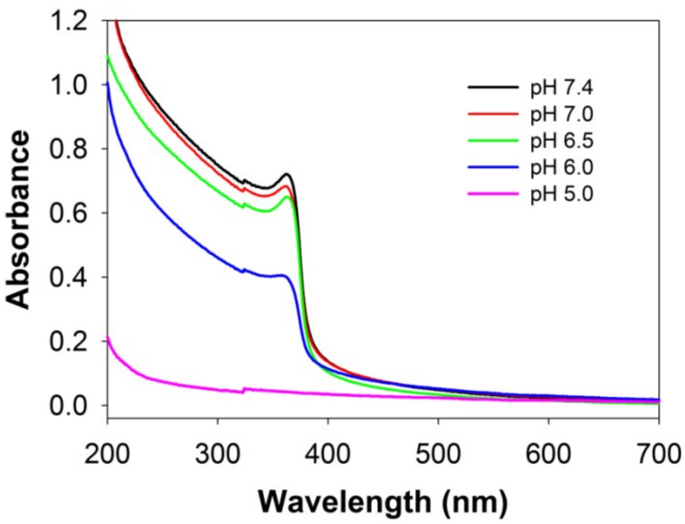

Fig. S6 UV-vis absorption spectra of aqueous solutions of ZnO NPs with a decreasing pH value from 7.4 to 5.0.

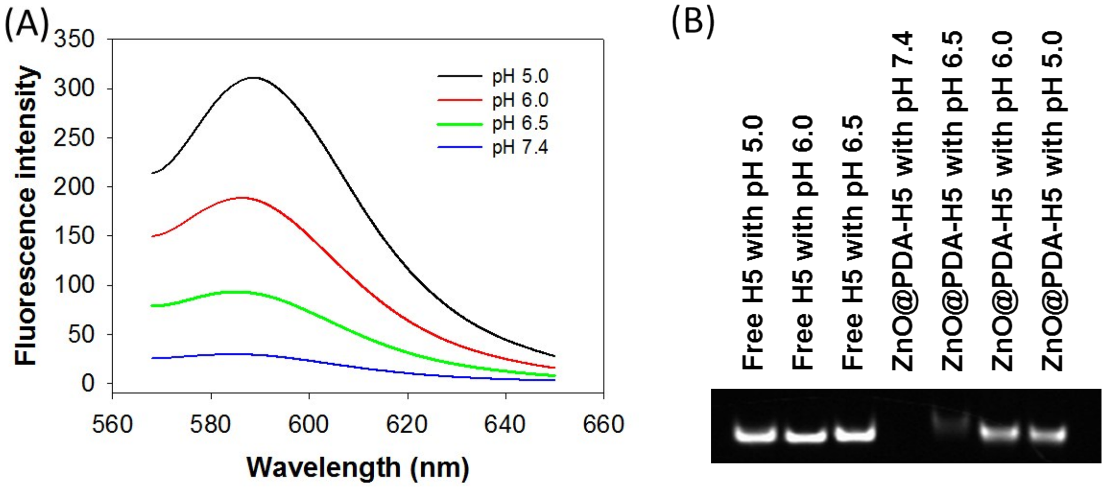

Fig. S7 The pH-triggered delivery of immobilized hpDNA on the ZnO@PDA NPs surface. (A) Fluorescence intensities of ZnO@PDA-H5 nanosystem solutions after 0.5 h of incubation under different pH conditions, (B) Polyacrylamide gel electrophoresis analysis free H5 and ZnO@PDA-H5 nanosystem solution, after 0.5 h of incubation under different pH conditions, respectively.

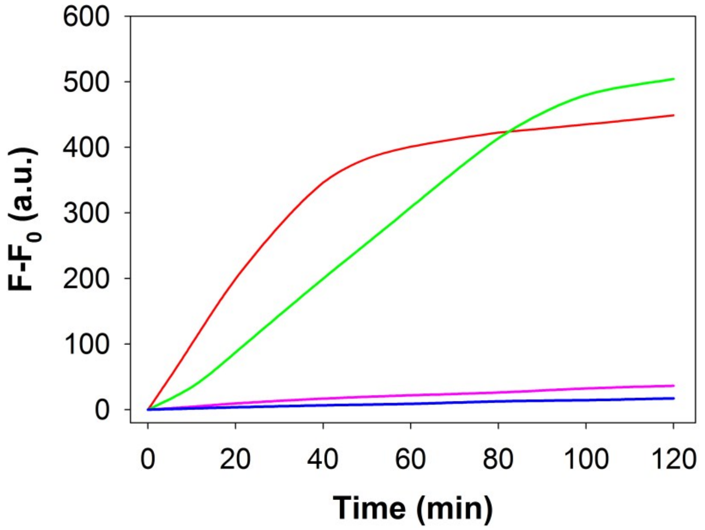

Fig. S8 Real-time fluorescence signals of ZnO@PDA-hpDNAs nanosystem carrying H0, H1, H2 and H3 in response to 20 nM target sequence (green), ZnO@PDA-hpDNAs nanosystem carrying H0, H1, H2 and H3 in response to 20 nM nontarget sequence (pink), ZnO@PDA-hpDNAs nanosystem carrying H1, H2 and H3 in response to 20 nM target sequence (blue), and free functional hpDNA

probes H0, H1, H2 and H3 with 20 nM target sequence (red).

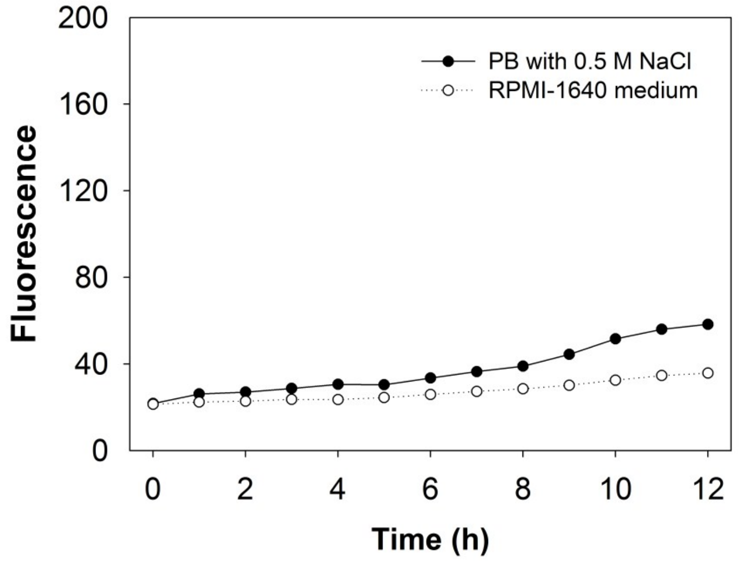

Fig. S9 The stability of ZnO@PDA-H4 nanosystem in RPMI 1640 medium and high salt (500 mM NaCl).

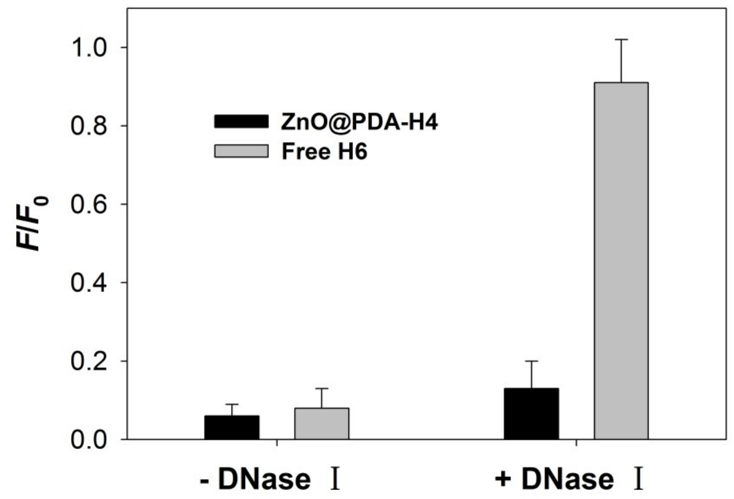

Fig. S10 Fluorescence assays for stability of hpDNA probes against DNase I. The fluorescence intensity ratios at wavelength of 526 nm for DNase I-mediated digestion reaction for ZnO@PDA-H4 nanosystem and free fluorescence self-quenching probe H6.

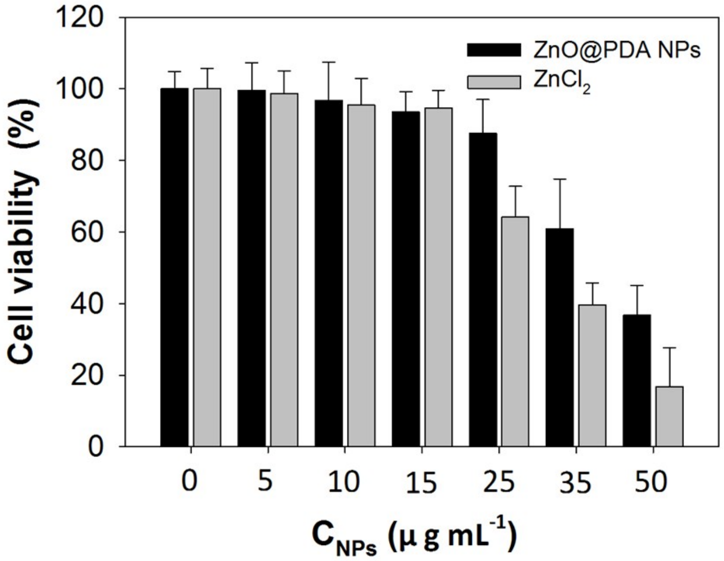

Fig. S11 The cytotoxicity of ZnO@PDA NPs and comparable concentrations of Zn^{2+} ions. The cell viability values (%) are determined by the incubated HeLa cells with nanoparticles of varying concentrations (0, 5, 10, 15, 25, 35, 50 μg mL^{-1}) for 48 h.

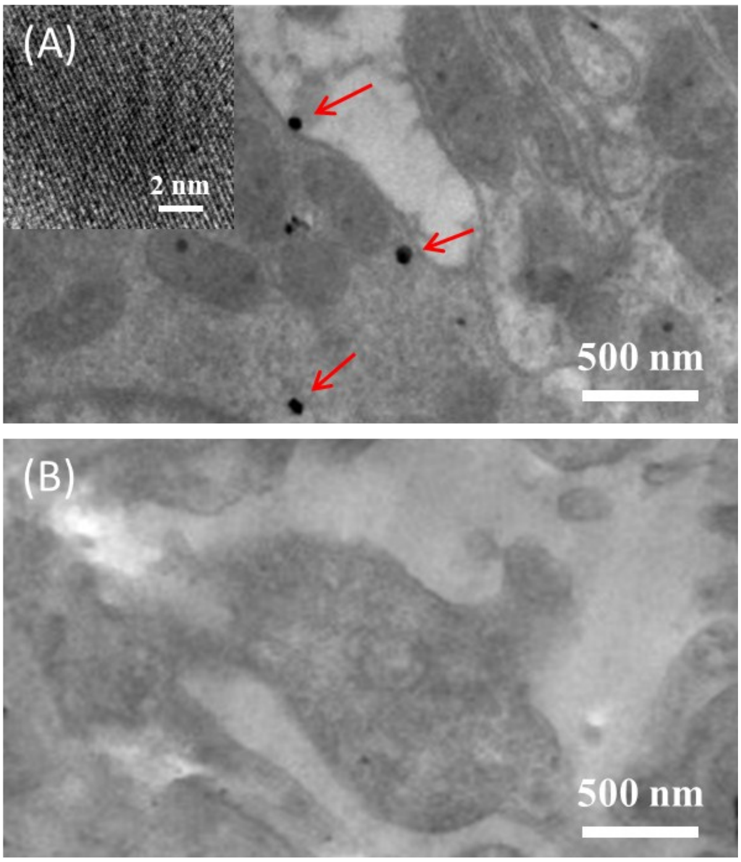

Fig. S12 Bio-TEM images of HeLa cells with ZnO@PDA-H7 nanosystem before (A) and after being incubated with nanosystem-free culture for 10 h (B). Inset: the crystal structure of ZnO NPs (red

arrows) in the cytosol.

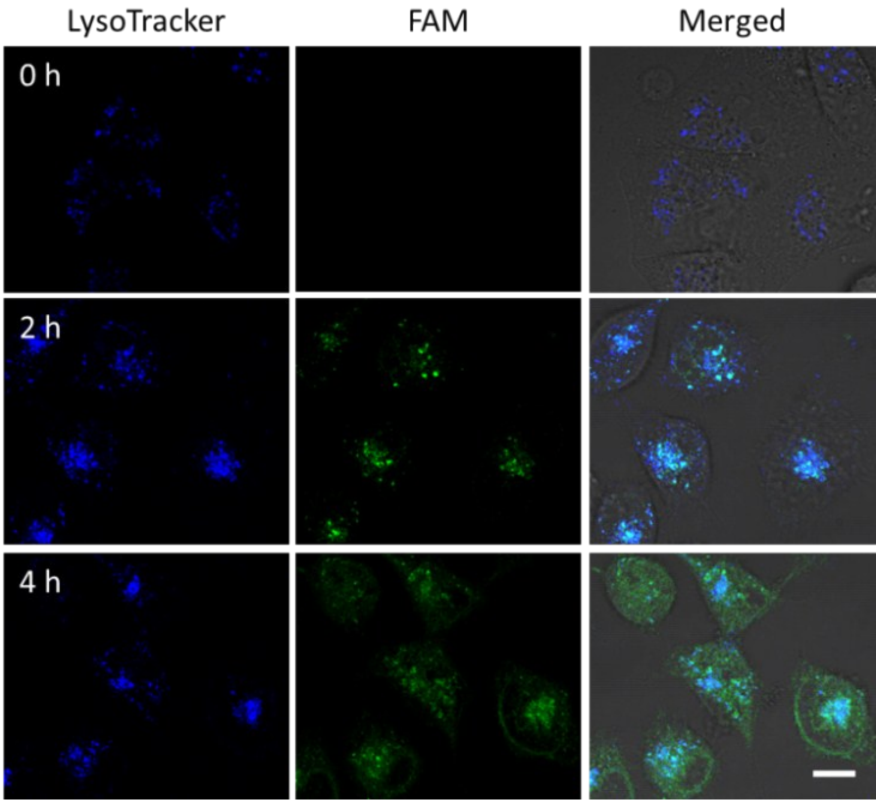

Fig. S13 CLSM images of HeLa cells after being incubated with ZnO@PDA-H7 nanosystem for 0, 2, and 4 h. The lysosomes of cells were stained with LysoTracker Blue (blue). Scale bar = 25 μm.

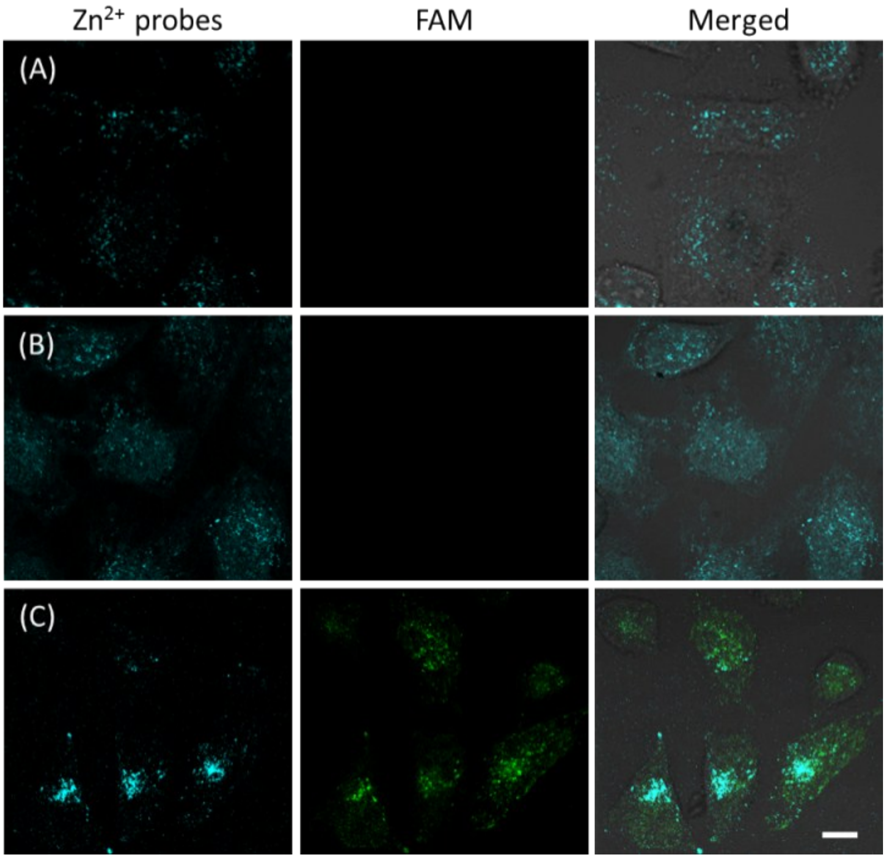

Fig. S14 CLSM images of HeLa cells after being incubated with A) no agents, B) $ZnCl_2$, and C) ZnO@PDA-H7 nanosystem for 12 h. The Zn^{2+} ions were stained with fluorescent Zinquin ethyl ester (cyan). Scale bar = 25 µm.

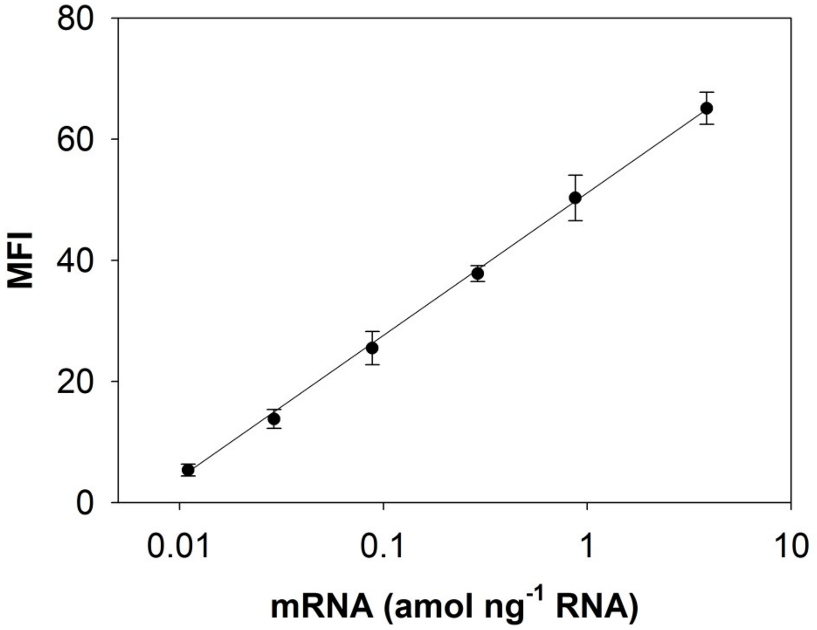

Fig. S15 Plot of MFI of cells versus intracellular quantity of *survivin* mRNA.
